# The Neuropathic Itch Caused by Pseudorabies Virus

**DOI:** 10.3390/pathogens9040254

**Published:** 2020-03-31

**Authors:** Kathlyn Laval, Lynn W. Enquist

**Affiliations:** Department of Molecular Biology, Princeton University, Princeton, NJ 08540, USA; lenquist@princeton.edu

**Keywords:** Pseudorabies virus, neuropathic itch, immunopathogenesis, neuropathogenesis, swine, non-natural hosts, neuroinflammation

## Abstract

Pseudorabies virus (PRV) is an alphaherpesvirus related to varicella-zoster virus (VZV) and herpes simplex virus type 1 (HSV1). PRV is the causative agent of Aujeskzy’s disease in swine. PRV infects mucosal epithelium and the peripheral nervous system (PNS) of its host where it can establish a quiescent, latent infection. While the natural host of PRV is the swine, a broad spectrum of mammals, including rodents, cats, dogs, and cattle can be infected. Since the nineteenth century, PRV infection is known to cause a severe acute neuropathy, the so called “mad itch” in non-natural hosts, but surprisingly not in swine. In the past, most scientific efforts have been directed to eradicating PRV from pig farms by the use of effective marker vaccines, but little attention has been given to the processes leading to the mad itch. The main objective of this review is to provide state-of-the-art information on the mechanisms governing PRV-induced neuropathic itch in non-natural hosts. We highlight similarities and key differences in the pathogenesis of PRV infections between non-natural hosts and pigs that might explain their distinctive clinical outcomes. Current knowledge on the neurobiology and possible explanations for the unstoppable itch experienced by PRV-infected animals is also reviewed. We summarize recent findings concerning PRV-induced neuroinflammatory responses in mice and address the relevance of this animal model to study other alphaherpesvirus-induced neuropathies, such as those observed for VZV infection.

## 1. History of Aujeszky’s Disease 

In 1813, a disease in cattle characterized by intense itching was first described in the USA. A similar disease appeared in Switzerland in 1849 and was mistaken for rabies because of the similar symptoms observed in cattle and dogs. In 1902, Aladár Aujeszky, a Hungarian veterinarian, first demonstrated the infectious nature of the agent and was able to distinguish the disease from rabies after experimental inoculation of rabbits with tissue suspensions from a diseased ox, a dog, and a cat. The infected rabbits showed excitation and nasal pruritus followed by convulsions and died within 60 h post-inoculation (hpi) [1]. Thus, the disease became known as “Aujeszky’s disease” (AD). In 1910, Schmiedhoffer confirmed that the disease was caused by a virus by performing filtration experiments [2]. From 1902 to 1930, only single outbreaks of AD were reported mainly in cattle and dogs in Hungary, Romania, France, Russia, and Brazil and the USA. At that time, the name “pseudorabies” was given to the disease in Europe because of its similarity to clinical rabies, while the term “mad itch” was mainly used in the USA. In 1931, Shope finally reported that mad itch and pseudorabies were caused by the same virus [3]. Three years later, he demonstrated that the agent of “mad itch” in cattle was also present in swine [4]. He also noted that the disease spread in swine herd and that, if cattle were pastured in the same lot, transmission from swine to cattle took place through abrasion of cattle skin. Surprisingly, the disease was not transmitted from cow to cow. In 1931, the Netherlands was the first country where the virus was reported to be enzootic in pigs. In the following years, sporadic outbreaks of PRV in pigs occurred worldwide and pigs were identified as a reservoir for the virus. In 1934, Sabin and Wright reported that AD virus (ADV)/pseudorabies virus (PRV) was serologically related to herpes simplex virus (HSV), resulting in the classification of the virus into the herpesvirus group [5,6,7].

## 2. Case Reports of Neuropathic Itch Caused by PRV Infection in Non-Natural Hosts

Since the 1950s, PRV remained a major pathogen in swine due to the intensification of pig production worldwide, resulting in severe economic losses to the pig industry. Efforts have been directed primarily to eradicating PRV from pig farms by the use of effective marker vaccines, but few studies have focused on the pathogenesis of the mad itch [8]. For the most part, this is because the characteristic pruritus observed in PRV-infected cattle and other hosts is not a common clinical symptom in infected pigs. Although, in many European countries and in the United States, PRV has been eliminated from domestic pigs, the virus continues to circulate among wild boar and feral swine, which act as a reservoir for the virus. 

Many cases of PRV infection in non-natural hosts have been reported since the 1950s. In this review, we summarize all the major findings since 2004 (See Table 1). A total of 26 cases of PRV infection have been reported involving a total of 13 humans, 50 dogs, 2 cats, 6 cattle, 863 sheep, 3 goats, 3931 captive mink, 1 wild and 1200 captive foxes, 1 wolf, and 1 lynx. The case reports of PRV-infected animals occurred primarily in European countries, USA and China and resulted from direct contact with pigs while hunting or through the consumption of raw offal from infected pigs (wild and domesticated) or pig carcasses. The main characteristic clinical symptoms observed in all infected animals were severe pruritus in the head and neck areas accompanied by self-mutilation. All infected animals usually died within 24–48 h after the disease onset. Gross postmortem examination of all infected animals revealed purulent edema and inflammation of the subcutaneous tissues of the scratched area, hyperemia, and hemorrhages. On histopathological examination, perivascular infiltrates of neutrophils, lymphocytes, and a few macrophages were observed in the brainstem, medulla oblongata, meninges and trigeminal ganglia (TG). Neuronal degeneration and necrosis as well as gliosis, satellitosis, and the presence of intranuclear inclusion bodies within neurons, astrocytes and satellite cells were also noted. The presence of infectious PRV and PRV DNA was confirmed by virus isolation, immunohistochemistry (IHC) and polymerase chain reaction (PCR) on brain and TG samples obtained from dead animals.

In contrast, case reports for humans consisted mainly of patients hospitalized with fever, headaches, visual impairment, seizures, respiratory failure, and coma within 1–3 days after the appearance of the symptoms. No signs of pruritus with self-mutilation were observed. Brain magnetic resonance imaging (MRI) and computed tomography (CT) scans showed abnormal signals in the insular cortex, bilateral temporal lobes, and hippocampus. Patients were usually diagnosed with viral encephalitis with the exception of one who was diagnosed with endophthalmitis. All patients immediately received antiviral therapy (acyclovir) for approximately 2 weeks. Gross pathological examination was not applicable as all patients survived with the exception of one who died after failing to respond to antiviral treatment [9]. Unfortunately, an autopsy was not performed on this patient. The presence of PRV DNA was confirmed by PCR of the vitreous humor of one patient with endophthalmitis [10]. Next generation sequencing (NGS) was used to detect only a number of 2–74 unique sequence reads of PRV, and no other viruses, in cerebrospinal fluid (CSF) of patients. It was suggested that the use of antivirals before detection might be the main reason for the limited number of virus sequences detected. Serological tests confirmed the presence of PRV antibodies in the serum and CSF of those patients. However, the presence of PRV was never determined by virus isolation from vitreous humor or CSF samples to confirm its infectivity. Interestingly, all case reports in humans occurred in China and mainly resulted from eye and hand injuries of patients at work (pig farms or slaughterhouses). The high incidence of suspected human PRV infections identified here might to be related to the high prevalence of PRV in swine in China and repeated exposure to infected animal tissues [11,12]. Still, it is important to keep in mind that these incidences are not unique development within China or occur just recently, as previously described by [13].

The absence of pruritus in all infected patients resembles the clinical outcome of PRV infection in swine and may suggest important differences in the pathogenesis of PRV among humans, swine, and all other non-natural hosts. There is a strong evidence that PRV-induced neuropathology and the clinical outcome of infection are connected with the innate immune response. Still, it is not clear whether humans have developed a more controlled and effective immune response (similar to swine) against PRV infection than other non-natural hosts. In the following sections, we will discuss in more detail similarities and key differences in the neuropathogenesis and immunopathogenesis of PRV infections between non-natural hosts and pigs that might explain their distinctive clinical outcomes.

## 3. Pseudorabies Virus (PRV)

In this section, we provide basic information about PRV taxonomy and virion structure, which is useful to understand PRV pathogenesis. The replication cycle of PRV as well as the cell-associated spread of infection are not covered in this review as several excellent reviews already addressed these topics in detail [14,15,16].

PRV belongs to the family of the Herpesviridae, subfamily Alphaherpesvirinae, genus Varicellovirus. The virus is closely related to herpes simplex virus type 1 and 2 (HSV1 and HSV2) and varicella-zoster virus (VZV), causing cold sores, genital lesions, and chicken pox, respectively [17]. The virion is 150–180 nm in diameter and comprises four main structural components: Genome, capsid, tegument, and envelope. The viral genome consists of a linear double stranded DNA of approximately 150 kbp. The complete genome contains at least 70 open reading frames. The genome consists of a long unique region (UL) flanked by a short inverted repeat (TRL/IRL) linked to a short unique region (Us) flanked by an inverted repeat (TRS/IRS) [18,19,20]. The genome is enclosed in an icosahedral capsid, consisting of 162 capsomers. The genome and capsid together form the nucleocapsid that is surrounded by the tegument, a proteinaceous matrix that lines the space between the nucleocapsid and the envelope. Tegument proteins play an important role during virus entry, assembly, and egress [21]. The envelope consists of a bilayer of phospholipids derived from the trans-Golgi network of the host cell and contains different embedded glycoproteins. For PRV, 11 glycoproteins have been characterized (gB, gC, gD, gE, gG, gH, gI, gK, gL, gM, and gN) and named according to the nomenclature established for the related proteins of HSV-1 [15,16,22]. All glycoproteins are constituents of the virion, except gG, which is secreted into the medium by infected cells [23]. The envelope proteins play important roles in virion binding and entry, envelopment, egress, cell-to-cell spread, induction of protective immunity, and immune evasion [15,16].

**Table 1:** Case reports of neuropathic itch caused by pseudorabies virus (PRV) infection in non-natural hosts since 2004.

Case n°	Year of Occurrence	Country	Species and Number of Confirmed Cases	Source of Contamination	Characteristic Clinical Symptoms	Gross Pathology	Histological Findings	Death after Onset of Clinical Symptoms	PRV Detection	Publication
1	2004	Poland	Farm animals: 7 cattle, 3 goats, 3 sheep, 2 cats and 1 dog	Not clear	-**Local pruritus** with violent licking, chewing and rubbing of various body parts -Fever -Excessive salivation	Not specified	Not specified	48 h	Virus isolation and PCR on brain and internal organs samples	[24]
2	2006	Austria	1 hunting dog	Contact with feral swine while hunting	-**Severe pruritus** on the lip with self-mutilation -Fever -Tachypnea -Seizures	-Purulent edema of the lip -Hypertrophied heart and hyperemia	-Non purulent encephalitis with intranuclear inclusion bodies -Multifocal lymphohistiocytic perivascular infiltrate in the medulla oblongata -Multifocal necrosis ganglia	24 h	H&E staining, IHC, virus isolation and PCR on brain samples	[25,26]
3	2007	Belgium	2 hunting dogs	Eaten offal from a wild boar	-**Intense facial pruritus** with self-mutilation -Convulsions -Hypersalivation	Not specified	Not specified	24 h	Virus isolation and qPCR on brain samples	[27]
4	2008	Spain	30 minks	Eaten swine viscera incorporated to food mixture	Not specified	Hemorrhages, ischemia and systemic vasculopathy	Mild purulent ganglioneuritis and encephalomyelitis	48 h	IHC and PCR on oropharyngeal mucosa, TG, spinal cord and brain samples	[28]
5	2008–2010	Austria	6 hunting dogs	Contact to the shot boars	-**Intense pruritus** on the neck and shoulders with self-mutilation -Hypersalivation -Coma	-Itch associated lesions in the head area -Purulent inflammation and edema of the subcutaneous tissues	Non purulent encephalitis of the brainstem with intranuclear inclusion bodies	16–44 h	IHC and PCR on brain samples	[25,29]
6	2011	USA	3 hunting dogs	Contact with feral swine while hunting	-**Intense facial pruritus** with self-mutilation -Fever -Dyspnea -Vomiting -Muscle stiffness	Extensive subendocardial hemorrhage	Neutrophilic trigeminal ganglioneuritis	48 h	IHC and IF on TG samples	[30]
7	2011–2013	China	13 farm and pet dogs	Possible contact with infected pigs or consumption of raw meat	-**Pruritus** -Tachypnea -Dyspnea	Systemic hemorrhage	-Non suppurative encephalitis with severe perivascular cuffing and glia cell proliferation	Not specified	Virus isolation and PCR on brain samples	[31]
8	2012	Germany	1 hunting dog	Not specified	-Tremors -Trismus -Spasms of the musculature of the larynx and pharynx -Dyspnea -Vomiting	Not specified	Non suppurative encephalitis in the brainstem with perivascular cuffing of lymphocytes and macrophages	Not specified	IHC on TG and brain samples	[32]
9	2012	China	860 sheep	Vaccinated with live-attenuated PRV-Bartha K16 strain	-Intense rubbing and licking with self-mutilation in the area where the vaccine was injected -**Localized pruritus** -Fever -Paralysis -Dyspnea	None	Not specified	24 h	PCR and EM on brain samples	[33]
10	2010–2014	Italy	11 hunting dogs	Direct contact with infected pigs or fed with raw meat	-**Intense facial pruritus** -Tremors -Dyspnea	Acute pulmonary alveolar emphysema and edema	Not specified	24–48 h	IHC on brain samples	[34]
11	2014	Italy	1 wild fox	Contact with infected domestic swine/feeding on infected rodents	-**Head scratching** -Motor coordination -Rolling in the snow	-Subcutaneous edema (head) -Multiple skin abrasions from scratching	Not specified	48 h	Virus isolation on brain samples	[35]
12	2014	China	379 minks	Captive minks fed raw pork livers	-**Abdominal and facial pruritus** -Claw and cage biting -Dyspnea- Vomiting	-Systemic hemorrhage -Splenic lesions -Petechia and ecchymoses in the epicardia	Not specified	24–48 h	Virus isolation and IF on brain samples	[36]
13	2014	China	3522 minks	Fed raw pork meat?	-Pneumonia like symptoms -Diarrhea -Lethargy	Not specified	Not specified	48 h	Virus isolation and PCR on brain and internal organs samples	[37]
14	2014	China	1200 captive foxes	Fed raw pork livers	-Initial fever -**Intense pruritus** -Frequent snarling and repeated lying down and rising -Dyspnea -Vomiting	Not specified	Not specified	12–96 h	Virus isolation and PCR on brain samples	[38]
15	2014	USA	10 hunting dogs	Contact with feral swine while hunting and consumption of pig offal	-**Intense pruritus** with self-mutilation -Erythema -Vomiting	Not specified	Moderate lymphoplasmatic encephalitis in the brainstem	24 h	IHC, virus isolation and PCR on brain samples	[39]
16	2017	Spain	1 Iberian Lynx	Eaten raw pig meat or offal?	**Signs of scratching** in the neck with self-mutilation	-Congested meninges -Multifocal erosions of the duodenum	-Meningoencephalitis with neutrophil and mononuclear cell infiltration -Gastrointestinal tract lesions	Found dead	PCR and IHC on brain, tonsil and intestinal samples	[40]
17	2017	China	1 wolf	Fed pork or pig offal	-**Intense pruritus** -Paroxysmal convulsions -Quadriplegia -Dyspnea -Vomiting	-Hemorrhagic spots and necrosis in the liver -Hyperemia -Hemorrhages and edema in the meninges	Not specified	6 h	PCR on brain, tonsil and lung samples	[41]
18	2017	Serbia	1 domestic dog	Fed pig offal	-**Pruritus** in the head and neck -Ataxia	Focal pulmonary, gastric and renal hemorrhages	Not specified	24 h	Virus isolation and PCR on brain and internal organ samples	[42]
19	2017	China	1 human (female)	Eye contamination of pig sewage	-Fever -Headache -Visual impairment => Diagnosed with endophthalmitis => Under antiviral therapy (acyclovir)	Not applicable	Not applicable	The patient survived	NGS, Sanger sequencing and PCR on vitreous humor samples -PRV antibody test positive in CSF, 4 months after disease onset	[10]
20	2018	Argentina	1 domestic dog	-Exposure to a serologically positive swine farm -Direct contact with pigs or fed raw pork meat	-**Pruritus** -Tremors -Trismus -Spasms of muscles of the larynx and pharynx -Dyspnea -Vomiting	Not specified	-Mononuclear cell infiltration in meninges -Mild diffuse gliosis -Neuronal satellitosis in gray matter	24–48 h	Virus isolation and PCR on brain samples	[43]
21	2018	China	9 cattle	Close contact to pig house	-**Banging their head to the walls** (scratching) -Ataxia -Gait and salivation	Leptomeningeal hyperaermia	-Non suppurative meningoencephalitis with mononuclear perivascular cuffing -Neuronal necrosis -Satellitosis	24 h	PCR on brain samples	[44]
22	2018	China	1 human (male)	Veterinarian, hands punctured by a knife used during autopsy of dead swine	-Fever -Headaches -Seizures -Coma within 3 days after the appearance of symptoms -Lumbar puncture indicated an opening pressure -CT brain imaging showed hypodensity in the bilateral basal ganglia => Diagnosed with viral encephalitis => Under antiviral therapy for 2 weeks (acyclovir)	Not applicable	Not applicable	The patient survived	PRV gB (serum and CSF) and gE (serum) antibodies detected at day 21 and 28 after disease onset	[45]
23	2018	China	5 humans (4 males and 1 female)	-Contact with pigs at slaughterhouse -Hand injury at work	-Fever -Seizures -Respiratory failure -Visual impairment -Retinal necrosis -Brain MRI showed abnormal signs in the temporal lobes and insular cortex => All diagnosed with viral encephalitis => Under antiviral therapy (not specified)	Not applicable	Not applicable	All patients survived	NGS in CSF (2 to 37 PRV reads detected)	[46]
24	2019	China	1 human (male)	Contact with pigs at work (sick pig handler)	-Fever -Convulsions -Pulmonary inflammation -Visual impairment -Retinal necrosis -Brain MRI showed hypo-intensity in the bilateral temporal lobe and hippocampus => Diagnosed with encephalitis complicated with bilateral retinal necrosis => Under antiviral therapy (acyclovir)	Not applicable	Not applicable	The patient survived	NGS in CSF (72 PRV reads detected)	[47]
25	2020	China	1 human (male)	-Contact with pigs (pork vendor) -Hand cut	-Fever -Seizures -Coma within 1 day after the appearance of symptoms -Brain MRI showed abnormal signals in the bilateral frontal lobe, temporal lobe, insula lobe, basal ganglia and hippocampus -Inflammatory lesions in the bilateral hemisphere =>Diagnosed with viral encephalitis =>Under antiviral therapy for 2 weeks (acyclovir)	Not applicable	Not applicable	The patient survived	- NGS in CSF (74 reads detected) -PRV antibodies detected in plasma and CSF at 23 and 56 days after disease onset	[48]
26	2018	China	4 humans (3 male and 1 female)	-Exposed to raw pork at work -1 injured during pork cutting	-Fever -Seizures -Coma within 1 to 4 days -Respiratory failure -Brain MRI showed abnormal signals mainly in the bilateral areas of the temporal lobe and bilateral basal ganglia -2/4=> Bilateral retinitis => All diagnosed with viral encephalitis => Under antiviral therapy for 2 weeks (acyclovir)	Not applicable	Not applicable	Three patients survived and 1 died	-NGS in CSF -PRV antibodies detected in available serum of 3 patients	[9]

Central nervous system (CNS); Trigeminal ganglia (TG); Cerebrospinal fluid (CSF); Immunohistochemistry (IHC); Immunofluorescence (IF); Hematoxylin and eosin (H&E); Next generation sequencing (NGS); electron microscopy (EM); Polymerase chain reaction (PCR).

## 4. The Pathogenesis of PRV

Here, we compare the main steps in the pathogenesis of PRV between its natural host, the swine, and non-natural hosts that may inform the mechanisms governing PRV-induced neuropathic itch.

### 4.1. Introduction 

PRV is a highly contagious pathogen that causes respiratory disease, neurological disorders, and abortion in swine. Transmission occurs primarily through direct contact with oral and nasal secretions, but can also occur by aerosols, transplacentally or venerally [49,50,51]. Other animal species are commonly infected through direct contact with pigs or through the consumption of raw offal from infected pigs (See Table 1). The infected animals develop a severe pruritus and usually die within 2 days.

### 4.2. Primary Replication in the Upper Respiratory Tract (URT)

**Figure 1:**
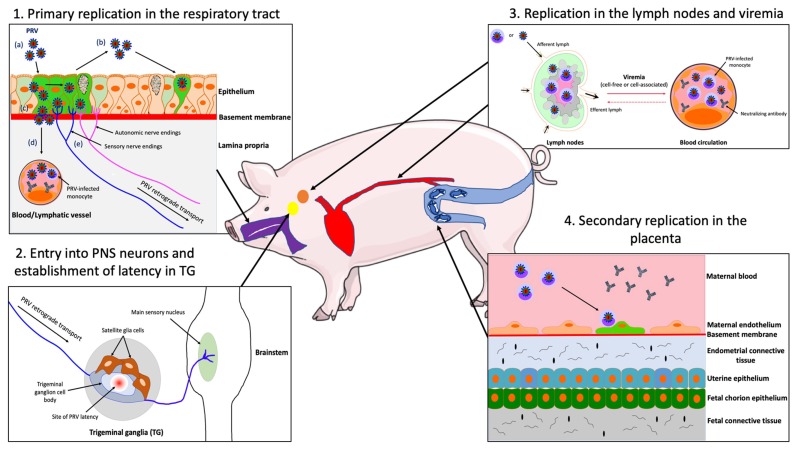
Schematic representation of the pathogenesis of PRV in the adult pig. (1) PRV first replicates in the epithelial cells of the URT; (**a**) PRV infection; (**b**) viral spread within the respiratory epithelium and viral shedding; (**c**) PRV breaches the basement membrane via protease activity and penetrates the lamina propria; (**d**) PRV reaches the draining lymph nodes and blood circulation; (**e**) PRV enters nerve endings of the PNS, including those coming from the trigeminal ganglia (TG) and spreads in the retrograde direction to the ganglia. (2) PRV establishes latency in TG neurons. (3) PRV replicates in the draining lymph nodes and establishes a cell-free and/or cell-associated viremia in PBMCs (mainly monocytes). (4) Via a cell-associated viremia in monocytes, PRV is transported to the placenta where it initiates a secondary replication in the endothelial cells lining the blood vessels of this organ. Purple = respiratory tract; red = blood circulation; orange = lymph nodes; yellow = TG; blue = uterus.

Upon entry into the natural host, PRV first forms foci of infection in the epithelial cells lining the URT, including nasal septum, tonsils, nasopharynx, trachea and lungs [14,52,53,54] (Figure 1(1a)). Ex vivo experiments showed that PRV-induced foci can be detected in the epithelium of swine nasal mucosa explants starting after 24 hpi [55,56]. Primary PRV infection of several tissues of the swine URT results in the destruction and erosion of the epithelium, causing mild respiratory symptoms, such as sneezing, coughing, dyspnea, and nasal discharge. These symptoms appear after 3 to 6 days and can last up to 10 days [51]. However, infected swine usually recover quickly, except for those who develop pneumonia due to secondary bacterial infections [57]. Viral shedding can be detected in nasal secretions from 1 to 14 days post-inoculation (dpi) [14,58] (Figure 1(1b)). 

**Figure 2:**
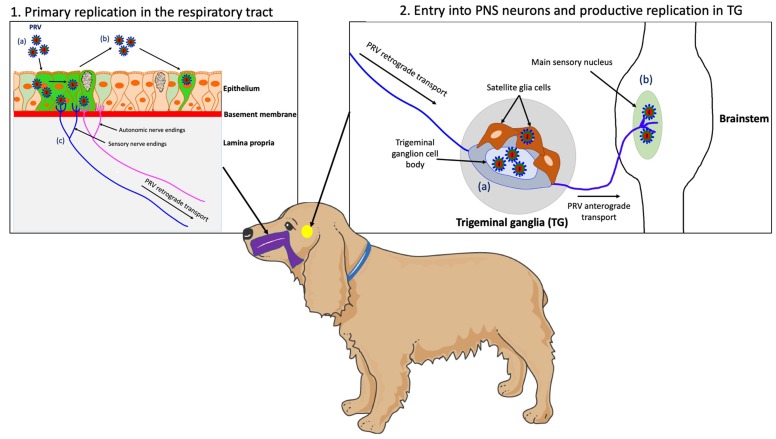
Schematic representation of the pathogenesis of PRV in a non-natural host, the dog. (1) PRV first replicates in the epithelium of the URT; (**a**) PRV infection; (**b**) viral spread within the respiratory epithelium and viral shedding; (**c**) PRV enters nerve endings of the PNS, including those coming from the TG and spreads in the retrograde direction to the ganglia. (2) PRV initiates a productive infection in TG neurons; (a) PRV replicates in cell bodies of TG neurons; (b) New progeny virions can further spread in the anterograde direction and infect the CNS (brainstem). Purple = respiratory tract; yellow = TG.

The entry of PRV into non-natural hosts has not been well studied but the presence, in most cases, of a pruritus in the head and neck dermatome, suggests that infectious virus particles mainly enter the body in natural conditions via the upper respiratory tract, including the nose, mouth, sinus, larynx and trachea (See Table 1 and Figure 2(1)). Experimental infection of mice via the intranasal route demonstrated PRV-positive cells in the respiratory epithelium at 6 and 24 hpi by immunofluorescence staining [7,59]. After intranasal inoculation, PRV was found in the nasal secretions of cattle and could be isolated from the nasal and pharyngeal mucosa and tonsils of infected animals [53] (Figure 2(1a)). Non-natural hosts can also shed PRV from nasal secretions, but only for a short time as the infected animals die very quickly (Figure 2(1b)). PRV also can replicate in the skin of mice after skin flank and footpad inoculations. Infectious virus was detected in the footpad around 18–24 hpi and in the skin of the flank area at 36 hpi [60,61,62]. 

### 4.3. PRV Entry into the Peripheral Nervous Ssystem (PNS) Neurons and Spread to the Central Nervous System (CNS)

After primary replication in the nasal epithelium of adult swine as described in 4.2, PRV enters nerve endings of the PNS, including those coming from the sensory TG and olfactory bulb as well as other facial, parasympathetic, sympathetic nerve neurons that innervate the nasal mucosa [59] (Figure 1(1e)). Virions travel via retrograde transport to the sensory and autonomic peripheral ganglia. A hallmark of herpesviruses is the establishment of a reactivable, latent infection in their hosts [63]. Accordingly, PRV establishes a lifelong latent infection in swine PNS neurons [64,65] (Figure 1(2)). Meanwhile, the infected pigs recover from the respiratory disease and become asymptomatic [58,66]. Upon stress-induced reactivation, viral replication occurs in the PNS ganglia, and virions spread in the anterograde direction along the nerves to the mucosal surfaces where the infection started [67]. Adult swine may exhibit mild respiratory signs upon viral reactivation. PRV rarely spreads in the retrograde direction to reach the CNS to cause encephalitis in adult swine. Cycles of latency and reactivation in pigs result in shedding of infectious virus and transmission to uninfected animals, facilitating the viral reservoir in herds. Interestingly, other human and animal herpesviruses, such as VZV and bovine herpesvirus type 1 (BHV-1) have been shown to invade PNS neurons in a similar way [68,69].

In non-natural hosts, PRV similarly enters nerve endings of the PNS that innervate the nasal mucosa and initiates a productive infection in PNS neurons (Figure 2(1c,2)). Infectious virus and DNA have been recovered from TG of naturally infected mink and dogs [28,30,32] (Figure 2(2a)). PRV particles were also found by electron microscopy in satellite glia cells and PNS neurons of autonomic ganglia from infected dogs after subcutaneous and intramuscular inoculations [70]. In contrast, a previous study from Fields and Hills described the absence of enveloped virions within satellite glia cells of lumbar dorsal root ganglia (DRG) after footpad inoculation, suggesting that an abortive infection occurred in these cells [62]. It remains unclear if PRV can productively infect satellite glia cells via transfer of virus particles from infected neurons. Following intranasal inoculation, PRV antigens were detected in the ipsilateral TG of mice at 3 dpi [59,71]. In dogs, PRV antigens were detected in stellate, celiac and caudal mesenteric ganglion at 4 dpi after subcutaneous inoculation [33]. Experimental inoculation of mice through the footpad and skin flank demonstrated the presence of infectious virus in the ipsilateral DRG starting from 42 and 36 hpi, respectively [61,62]. The presence of infectious virus suggests active viral replication in PNS neurons, which coincides with the start of pruritus in infected animals. As non-natural hosts die very quickly after PRV infection, it is not clear whether the virus is able to establish latency in PNS neurons. A study from Osorio and colleagues reported successful establishment of PRV latency in TG after passive immunization of mice prior to inoculation with an attenuated PRV strain (PRV-Bartha) [66]. The authors could recover infectious virus from TG explants and detected PRV DNA in latently infected TG in mice by PCR and in situ hybridization after 2 to 8 months post-inoculation. As of now, no studies have demonstrated the establishment of latency in non-natural hosts following inoculation with a virulent PRV strain that causes pruritus.

After replication in the PNS, progeny virions may spread in the retrograde direction from the PNS to the CNS if the animals survive long enough. Infectious virus and viral DNA have been recovered from brain samples of many naturally infected animals (See Table 1 and Figure 2(2b)). PRV replication in the brain causes encephalitis. After intranasal inoculation, the virus was detected in the mouse brainstem at 3 dpi [71]. In addition, infectious PRV was detected in the brainstem of infected mice around 66–72 hpi following skin flank and footpad inoculations [61,62]. PRV DNA was also detected in the spinal cord and hindbrain of infected mice at 82 hpi (moribund state) following footpad inoculation. Interestingly, the midbrain and forebrain were rarely found positive for viral antigens, probably due to the short survival of the animals. These findings suggested that extensive viral replication in the brain is not responsible for the acute death of the infected animals.

### 4.4. PRV Replication in the Draining Lymph Nodes and Viremia

Following replication in the swine respiratory epithelium, PRV may cross the basement membrane (BM) in foci to penetrate the connective tissues and reach the draining lymph nodes and bloodstream [56,72] (Figure 1(1c,d)). The invasion of PRV through the BM towards the lamina propria is mediated by trypsin-like serine protease activity [73]. Within 24–48 hpi, infectious virus as well as viral antigens can be detected in swine inguinal lymph nodes [74,75,76]. Virus can persist in pharyngeal lymph nodes for up to 35 days [77]. PRV infection is amplified in the draining lymph nodes with discharge of infected leukocytes, via the efferent lymph, into the blood circulation. As a result, PRV initiates a cell-associated viremia in peripheral blood mononuclear cells of swine (mainly monocytes) that facilitates dissemination within the host (Figure 1(3)). Cell-free viremia can also occur in infected pigs [78,79]. Viremia can be detected after 1 dpi and persists for 2 weeks. The cell-associated viremia is a prerequisite for the dissemination of PRV to the pregnant uterus.

There are only a few studies that confirmed the presence of infectious PRV in lymph nodes of non-natural hosts following experimental infections. In cattle, PRV could be isolated from the retropharyngeal lymph nodes following intranasal inoculation [53]. Virus was also detected in the pituitary, pharynx and submaxillary lymph nodes of cattle after oral infection [80]. However, PRV was not detected in the lymph nodes of experimentally and naturally infected dogs [30,33]. So far, no evidence exists for cell-free or cell-associated viremia in non-natural hosts. No infectious virus was detected in tissues other than the nervous system of infected dogs and mice [30,33,81]. In addition, intravenous inoculation of cattle with PRV failed to disseminate the virus throughout the body. Consequently, these results suggested that a viremia is unlikely to occur in non-natural hosts.

### 4.5. Secondary Replication in the Swine Pregnant Vterus 

Once in the blood circulation, infected monocytes adhere to and subsequently transfer PRV to the endothelial cells (EC) lining the blood vessels of the placenta (Figure 1(4)). Secondary replication in the placenta can cause vasculitis and multifocal thrombosis, resulting in abortion [82,83,84]. The infection of EC is mediated by cell-to-cell contacts between infected monocytes and EC [85]. The development of abortion may depend on the hormonal activity and immune status of sows during pregnancy. Indeed, it was demonstrated that the expression of adhesion molecules on EC is induced by cytokines and hormones present in the local environment during pregnancy [86,87,88]. These cytokines may facilitate the adhesion of infected monocytes to the endothelium.

After intranasal, intra-uterine, and intra-fetal inoculations of vaccinated pregnant sows, PRV antigen can be detected in vaginal and sacral ganglia [89]. A widespread EC infection may cause detachment of the fetal membranes during the first trimester of gestation, thus leading to the abortion of a virus-negative fetus or cause fetal reabsorption in swine. Less extensive uterine vascular pathology may allow for transplacental infection and lead to the abortion of a virus-positive fetus during the second and third trimester of gestation or stillborn pig [51,90]. Viral aborted fetuses usually show multiple lesions, including foci of necrosis in the liver, spleen and lungs. PRV can be isolated from the liver, spleen, lungs, body fluids, and brain from fetuses aborted from vaccinated pregnant sows [82,91]. 

At this time, no cases of abortion have been described in pregnant cows, dogs or other non-natural hosts after PRV infection. After primary replication in the nasal epithelium, a secondary replication of PRV in the placenta of these animals is unlikely due to the absence of a cell-associated viremia.

### 4.6. PRV Infection in Suckling and Weaned Piglets

PRV infection is more severe in piglets than in adult swine [53]. In neonates, sudden death usually occurs with few to no clinical signs. In suckling pigs, death is preceded by fever, vomiting and CNS signs such as problems of coordination, weakness of the hindquarters, convulsions and paralysis. Mortality in neonates and suckling pigs is close to 100%. In weaner pigs, clinical signs resemble those in suckling pigs with the involvement of respiratory signs including dyspnea, sneezing, coughing, and nasal discharge. Mortality rate is around 5–10%. No marked pruritus develops in pigs of any age. Infectious virus could be detected in brain samples of four piglets naturally infected with PRV [92]. The severity of the symptoms decreases with age and has been correlated to a more effective immunity in adult swine compared to that of piglets.

## 5. The Pathogenesis of PRV-Induced Neuropathic Itch

In this section, we will briefly introduce the definition and clinical classification of itch and further dissect the neuronal and immunological basics for the unstoppable itch sensation in PRV-infected animals.

### 5.1. Clinical Classification of Itch

Itch, also known as a pruritus, was defined in 1660 by a German physician Samuel Hafenreffler as an “unpleasant sensation that elicits the desire or reflex to scratch”. Pruritus can be acute or chronic [93,94]. Acute itch can be triggered by insect bites and relieved by scratching. Scratching, in turn, generates a mild pain that inhibits the itch sensation. In contrast, chronic itch can last for a longer period of time (>6 weeks duration) and scratching usually does not relieve the itch [95,96]. 

A clinical classification of chronic itch has been proposed by the International Forum for the Study of Itch and comprised 4 categories: the pruriceptive itch, the neurogenic itch, the neuropathic itch and the psychogenic itch (IFSI; http://www.itchforum.net). The first category is the pruriceptive itch that is caused by inflammatory skin disorders, such as atopic dermatitis, psoriasis, drug reactions, mites, and uticaria [97]. Pruriceptive itch originates following activation of primary afferent nerve terminals located in the skin. The main pruritogens, or itch-producing stimuli are histamine, interleukins, prostanglandins, and proteases. The second category is the neurogenic itch that results from CNS activation without necessary activation of sensory nerve fibers and is usually accompanied by visceral diseases such as chronic liver disease and chronic renal failure [98]. The third category is the neuropathic itch, a chronic condition that arises from viral-induced disease and/or traumatic nerve injury of the PNS or CNS, such as peripheral neuropathies (e.g., post-herpetic itch), multiple sclerosis and nerve compression or irritation (e.g., notalgia paresthethica, and brachoradial pruritus [99]. The main mediators of the neuropathic itch are neuropeptides, proteases and inflammatory mediators such as cytokines. Finally, the fourth category is the psychogenic itch related to psychological or psychiatric disorders, such as itch associated with delusions of parasitosis, stress and depression [100].

### 5.2. The Neuropathic Itch

Neuropathic itch (NI) is defined as perception of itch in the absence of pruritogenic stimuli [101]. NI can originate at any point along the sensory afferent pathway as a result of damage of the PNS and less frequently of the CNS. These PNS lesions occurs in sensory itch neurons including slow conducting myelinated (Aδ) and unmyelinated (C) nerve fibers [102]. In contrast, lesions that affect motor neurons are not associated with NI. NI are often characterized depending on the location of the nerve damage. For instance, brachioradial pruritus and notalgia paresthetica are focal NI, caused by damage of small fibers within cervical spinal nerves and damage to the cutaneous branches of the posterior divisions of the spinal nerves, respectively [103]. The most common focal NI arising from sensory ganglia lesions occurs during VZV reactivation within sensory ganglia, initially presenting as the zosteriform lesion known as shingles. In contrast, polyneuropathies arise from generalized peripheral nerve damage [104]. Finally, NI syndromes can also arise from lesions within the spinal cord (e.g., multiple sclerosis) and in the brain (stroke) [105,106,107]. In the brain, any types of lesions that damage itch circuitry can cause NI.

The mechanisms underlying the neuropathic itch are still poorly understood and data are scarce. The main consensus is that peripheral nerve injury activates PNS sensory itch neurons to fire excessively and thus, stimulate excitative interneurons in the dorsal horn of the spinal cord to release gastrin-releasing peptide (GRP). Then, the release of this neuropeptide further stimulates spinothalamic tract (STT) neurons that send itch signals to the thalamus [108,109]. Finally, these signals are relayed and processed in the somatosensory cortex. The central inhibition of itch pathway neurons in the brain that should be in turn activated, is likely dampened or disabled, resulting in an unstoppable itch sensation [110].

### 5.3. The Neuronal Mechanisms of PRV-Induced Neuropathic Itch

Based on studies of the pathogenesis of PRV in non-natural hosts (see Section 4.3), continuous PRV replication in PNS ganglia causes neuronal lesions that are likely responsible for the initiation of the pruritus in these animals. Several animal models have been used to further dissect the mechanisms by which PRV replication in PNS neurons cause pruritus. 

A study from Dempsher and colleagues first demonstrated that PRV induces spontaneous, intermittent discharge of nerve impulses over the preganglionic and postganglionic nerves of superior cervical ganglia following ocular inoculation. The spontaneous discharges were only found in PRV-infected sympathetic ganglia of rats showing pruritus [111]. Similar results were observed after intraocular and intradermal PRV inoculations in rats [112,113,114]. Interestingly, inoculation with a PRV pruritus-producing strain (L strain) induced spontaneous hyperexcitability of neurons. In contrast, the non-pruritus producing strain (G strain), known to cause meningoencephalitis, exhibited impaired sympathetic synaptic conduction in infected rats [112]. Voltage-gated sodium and calcium channels were found to be responsible for the initiation and propagation of action potential (AP) in the infected ganglia [114]. 

In addition, PRV infection induces electrical coupling and increases AP firing rates in cultured rat sympathetic neurons in vitro [115]. The formation of fusion pores between infected PNS neurons was found to be mediated by PRV gB. PRV gB protein is an important component of the viral membrane fusion complex (gB/gH/gL) and is crucial for viral entry into neurons [116]. The production of fusion pores facilitates the flow of ions between PNS neurons and causes direct electrical coupling [115]. Moreover, it was demonstrated that infection of PNS neurons of the submandibular ganglia with a virulent PRV pruritus-producing strain (PRV-Becker) induces synchronous and cyclical activity in neuronal cell bodies [117]. Also, it was found that newly made virus particles in infected neurons were transported in axons back to the glands where the infection started. Thus, the authors introduced the concept of “round-trip” reseeding and amplification of the infection in the ganglia. The ability to reseed the gland increases the infection of the innervating ganglia and the involvement of more axons in electrical firing, therefore directly contributing to the pruritus. In contrast, mice infected with an attenuated, live vaccine and non-pruritus producing strain (PRV-Bartha) did not show signs of synchronous and cyclical activity in infected ganglia. This difference was attributed to the fact that PRV-Bartha lacks the US9 protein required for sorting virion proteins into axons [118]. Recent results are in agreement with the PRV round-trip concept. For instance, a large amount of infectious virus was detected in the mouse footpad at moribund state after footpad inoculation. Likely, it resulted from virus particles that originally infected the DRG, replicated and went back to the footpad rather than from local viral replication in the footpad. Indeed, the inoculated footpad, which exhibited epidermal necrosis accompanied by immune cell infiltrates, did not have time to regenerate and support efficient viral replication [81]. 

Finally, a comparative study of the neuroinvasive mechanisms between virulent PRV-Becker and attenuated PRV-Bartha was performed using the mouse flank inoculation model. In contrast to PRV-Becker infection, mice infected with PRV-Bartha did not develop pruritus and lived twice as long. However, they did show severe CNS symptoms due to widespread PRV-Bartha infection in the brain and eventually died of viral encephalitis. Using several PRV mutants, the authors demonstrated that the pruritus stimulus was mainly mediated by US9, gE and gI proteins [61]. These 3 gene products, which are deleted in the PRV-Bartha strain, are required for virulence and efficient anterograde spread of PRV within the nervous system [118,119,120]. 

### 5.4. The Immune Mechanisms of PRV-Induced Neuropathic Itch

The immune system plays a crucial role in the development of neuropathic itch. In the case of a pruriceptive itch, skin inflammation results in the recruitment and activation of immune cells to the skin epithelium. Activated immune cells release pro-inflammatory mediators, such as interleukin (IL) 31 and IL-33 that sensitize pruriceptors, leading to peripheral sensitization and activation of itch signaling pathways [121]. In the case of a neuropathic itch, peripheral nerve injury can cause inflammation of the nervous system, so called neuroinflammation. The PNS and CNS neurons as well as resident satellite glia, microglia, and astrocytes can also produce inflammatory mediators, including pro-inflammatory cytokines and chemokines, neuropeptides and reactive oxygen species. The release of neuropeptides such as substance P and calcitonin gene-related peptide (CGRP) by activated primary sensory neurons has been shown to have paracrine effects on immune cells and can increase the inflammation and subsequently amplify the itch sensation [122,123]. The same localized immune activation can be mimicked after viral infection of PNS neurons. For instance, reactivation of VZV from sensory ganglia causes a self-limited dermatomal rash with pain and itching, which is accompanied by inflammation of the skin [124]. 

**Figure 3:**
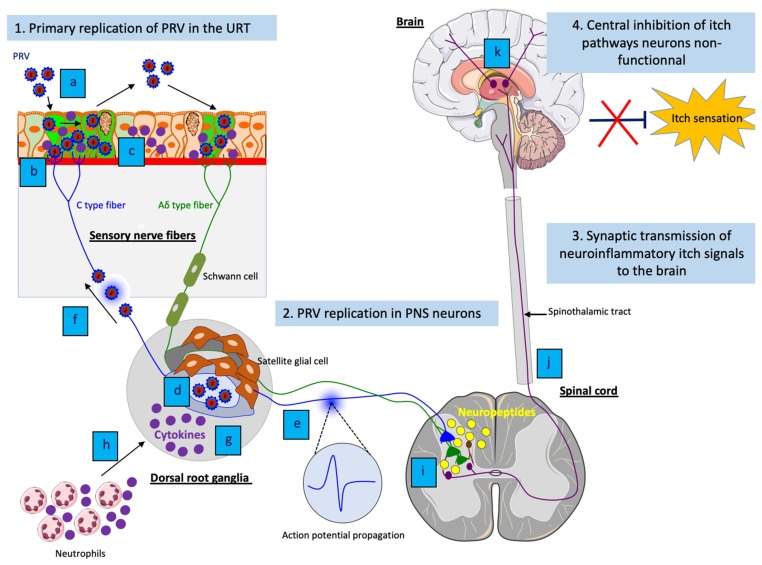
Model of PRV-induced neuropathic itch in non-natural hosts. (1) (**a**) Efficient viral replication and spread of PRV in the respiratory epithelium; (**b**) PRV particles activate axons terminals of sensory PNS neurons; (**c**) Activated sensory PNS neurons trigger inflammatory signaling pathways and produce pro-inflammatory cytokines that are released in PNS neurons and locally at axon terminals. (2) (**d**) Efficient PRV replication in cell bodies of PNS neurons and release of new progeny virions; (**e**) Spontaneous hyperexcitability of neurons and increase of action potential firing as well as (**f**) reseeding of new progeny virions back to the epithelium increases electrical coupling of axons and contributes to the pruritus; (b) Amplification of the inflammatory response in PNS neurons; (**h**) The production of pro-inflammatory cytokines in PNS neurons attract neutrophils and other immune cells to the site of infection and propagate the neuroinflammation. (3) (**i**) Release of neuropeptides from activated PNS neurons stimulate excitative interneurons in the dorsal horn of the spinal cord; (**j**) The excitation spreads to spinothalamic tract neurons, which in turn send neuroinflammatory itch signals to the brain; (**k**) These signals are relayed and processed in the somatosensory cortex. (4) The central itch inhibition pathways are likely dampened or disabled, resulting in an unstoppable pruritus.

Several cases of PRV-induced neuropathic itch reported mild purulent ganglioneuritis and encephalomyelitis in non-natural hosts, thus suggesting that PRV infection of sensory ganglia is accompanied by a specific inflammatory response (See Table 1). Indeed, perivascular cuffing of lymphocytes, monocytes and macrophages as well as a neutrophilic cell infiltration were detected in PRV-infected ganglia. So far, only a few studies investigated the role of the inflammatory response in the initiation and development of PRV-induced neuropathic itch. By the use of the mouse flank inoculation model, PRV-Becker infected mice, that were anesthetized at the time the pruritus started, did not develop skin lesions. Still, these mice died as the same time as the non-anesthetized ones. These results suggested that self-mutilation and scratching alone were not the cause of death. The authors then mentioned that a peripheral host immune response to PRV infection of the PNS could be an important factor in the death of the infected animals [61]. Using the footpad inoculation model, it was later shown that virulent PRV-Becker, but not attenuated PRV-Bartha, infection induces a specific and lethal systemic inflammatory response in mice. High levels of IL-6 and granulocyte colony-stimulating factor (G-CSF) were measured in both tissues and plasma of infected animals, including the footpad and DRG at moribund stage [81]. Furthermore, a strong correlation was found between the level of infectious virus detected in the DRG and footpad and the production of pro-inflammatory cytokines. Indeed, PRV-Becker replicated to a higher level in both tissues than PRV-Bartha. The fact that PRV-Becker was able to reseed new progeny virions back from the DRG neurons to the footpad might also have contributed to the amplification of the inflammatory response. Figure 3 shows a model of PRV-induced neuropathic itch in non-natural hosts.

Both IL-6 and G-CSF are produced by immune cells (neutrophils, T lymphocytes and macrophages) and neurons. While IL-6 has pleiotropic effects on immune response, inflammation, hematopoiesis and neurogenesis, G-CSF is mainly a key regulator of neutrophil function, mainly influencing the migration of neutrophils across the vascular endothelium [125,126,127]. Taken together, the high concentrations of G-CSF and IL-6 detected in the infected footpad and DRG of experimentally infected mice are likely to correlate with histological findings from naturally infected animals where a massive neutrophilic infiltration is observed in the PRV-infected ganglia. Furthermore, neutrophils can induce neurotoxicity on DRG neurons and are considered responsible for hypersensitivity and neuropathic pain observed after peripheral nerve injury [128]. Therefore, their accumulation around the infected ganglia may further amplify the neuroinflammation. 

The early events of the neuroinflammatory response of PRV infection in mice were recently characterized. Using the mouse footpad inoculation model, it was demonstrated that PRV-Becker infection primes DRG neurons to a state of inflammation very early post-infection [60]. More specifically, the authors found that the peak of IL-6 and G-CSF production detected in the DRGs and footpad of infected mice at 7 hpi could not be attributed to the infiltration of neutrophils in these tissues that occurred at 82 hpi. An efficient replication of PRV-Becker and subsequent spread in the footpad were necessary to activate DRG neurons to produce G-CSF at a very early time pi. Moreover, PRV replication was limited in the footpad of Toll-like receptor 2 (TLR2) knockout (KO) mice with no viral replication detected in DRG neurons. TLRs are expressed in nociceptive neurons and play a crucial role in neuroinflammation [129,130]. In particular, TLR2 is responsible for the activation of spinal cord glial cell after nerve injury and subsequent pain hypersensitivity [131]. Thus, the results suggested that TLR2 might be a potential receptor for PRV on DRG neurons, thus facilitating viral spread and the initiation of the neuroinflammatory response in mice. PRV gB expressed on new progeny virions or infected epidermal cells was proposed as a potential candidate to interact with TLR2 expressed on axon terminals of DRG neurons that are innervating the footpad.

### 5.5. Why PRV-Infected Swine Do Not Itch

As described in Section 4, the pathogenesis of PRV is very different between its natural host, the pig, and other mammals. One first essential difference is the establishment of latency in PNS neurons of pigs followed by sporadic periods of reactivation. In contrast, PRV productively replicates in PNS neurons of non-natural hosts following primary infection of the URT and the infection can spread further to the CNS. A latent infection is not established. The second main difference is that productive PRV infection of PNS neurons triggers a specific inflammatory response that contributes to the initiation and development of the pruritus in non-natural hosts.

Using compartmented rat neuronal cultures, it was demonstrated that efficient PRV retrograde transport in axons depends on the number of virus particles infecting axons. A threshold for the number of infecting particles in axons is set for establishing a quiescent (below a MOI of 0.1) or productive (above a MOI of 0.1) infection in rodent PNS neurons [132]. A similar threshold of PRV infection may exist in pigs. Better immune control of the infection at the primary site, the URT, may limit the number of PRV particles reaching the cell bodies of swine PNS neurons facilitating the latent infection. 

Interestingly, type I IFN suppresses PRV production replication in porcine TG neurons and porcine epithelial cells in vitro [133,134]. All PRV-infected TG neurons were in a stably suppressed quiescent state of infection after type I IFN pretreatment. More specifically, it was demonstrated that type I IFN decreases PRV IE180 protein expression level in sensory neuronal cells, allowing for the establishment of a quiescent state [135]. Pretreatment of axons with type I IFN significantly reduced the number of PRV particles moving in axons towards the cell bodies of rat PNS neurons [136]. In addition, Lamote and colleagues demonstrated that PRV-Becker inhibits the IFN response in swine dendritic cells while PRV-Bartha induces a strong type I IFN response in these cells. This difference was attributed to the absence of the immunomodulatory glycoprotein gE/gI gene complex, in the genome of Bartha [137]. So far, the level of type I IFN has not been quantified in the swine URT and compared between PRV-Becker and PRV-Bartha infections. Therefore, it is not known whether the PRV gE/gI complex similarly inhibits the type I IFN response in the swine respiratory epithelium. If so, additional antiviral factors, other than the induction of a strong type I IFN response, may exist further restricting primary PRV infection in pigs. For instance, a group of antiviral restriction factors, the IFN-inducible transmembrane proteins (IFITMs) have been shown to inhibit PRV replication in porcine kidney epithelial (PK-15) cells and porcine alveolar macrophages. Porcine IFTM1 specifically inhibited PRV entry in these cells [138]. Furthermore, a porcine IFN-stimulated ubiquitin-like protein (pISG15) efficiently inhibited PRV infection by reducing viral titers and increasing type I IFN expression in vitro [139]. 

In addition to its role in reducing viral replication, type I IFN plays a crucial role in regulating the early neuroinflammatory response and clinical outcome of PRV infection in mice [60]. In the footpad inoculation model, PRV-Bartha infection, but not PRV-Becker, induces a strong type I IFN response in the footpad and DRG neurons of inoculated mice, which in turn fails to trigger an inflammatory response in those tissues. Surprisingly, PRV-Bartha infection of type I IFN KO mice induces the production of pro-inflammatory cytokines, such as G-CSF in DRG neurons. Therefore, PRV-Becker infection may trigger a massive inflammatory response in mice because this virulent strain can suppress the IFN response. The imbalance of pro-inflammatory and antiviral immune responses might contribute to the distinct clinical outcomes of PRV-Becker and Bartha infections in mice. In addition, during PRV-Becker infection, the rapid priming of DRG neurons to an inflammatory state may initiate the neuropathic itch in mice as the infection of the PNS proceeds.

Taken together, these studies emphasize the essential role of the type I IFN response in controlling PRV replication in the swine respiratory epithelium. This control may prevent the productive PNS infection and the induction of a powerful inflammatory response, which may be the reason why infected pigs do not show itch symptoms.

## 6. PRV Infection in Mice: A New Animal Model for VZV-Induced Peripheral Neuropathies

A better understanding of PRV-induced neuroinflammatory responses in mice may provide new insights in the initiation and development of virus-induced neuroinflammation during other herpesvirus infections. For instance, this animal model could be useful to dissect the mechanisms of neuropathic itch in patients with post-herpetic lesions (e.g., herpes zoster (HZ); shingles). Indeed, the neuropathogenesis and immunopathogenesis of VZV and PRV infections are remarkably similar. Reactivation of VZV causes a self-limited dermatomal rash with pain and itching, which is accompanied by inflammation of the skin [124]. The HZ lesions can be reduced by treatment with antivirals [140]. However, postherpetic neuralgia (PHN) and postherpetic itch (PHI) are two common complications of HZ that can occur in some cases in up to 50% of patients with shingles [101,141].

PHN consists of a burning and stabbing pain while PHI is characterized by a relentless itch in the same area of the HZ rash, resulting in serious injuries due to scratching. Both PHN and PHI can last for months or years after resolution of the HZ rash, thus severely impacting the life quality of infected people. Antiviral therapy for acute HZ does not eliminate the risk of PHN, and no beneficial effect of any antiviral drug on established PHN has been shown [142]. It was suggested that PHN is caused by VZV-induced inflammation and axonal damage, which gives rise to hyperexcitability, marked by spontaneous firing of PNS neurons. These neurons may have a lower excitation threshold to pain, thus causing neuropathic pain [143,144]. In contrast, the underlying mechanism(s) of PHI is largely unknown. Despite some similarities between itch and pain pathways, treatments against pain are not efficient in relieving itch. Current treatments against neuropathic itch are very limited and lack specificity, and for many patients with PHI no alleviation can be provided [145]. 

The narrow host range and lack of clinical disease have limited the use of animal models to investigate the pathogenesis of VZV infection [146]. So far, investigation of PHI has been limited by the lack of a relevant in vivo neuropathic itch animal model. Interestingly, VZV and PRV infections present multiple similarities in genome sequence, clinical signs, pathogenesis and immunity. At the level of innate immunity, the exact same concentration of IL-6 (~30,000 pg/mL) has been demonstrated in both VZV-infected human explants and PRV-infected mouse footpad by ELISA [81,147]. Since VZV does not productively replicate in rodents, PRV-induced neuropathic itch in mice may represent a promising model to further understand the pathogenesis of PHI caused by VZV infection. 

## 7. Conclusions

Since the first case of mad itch was described 207 years ago, the characteristic pruritus caused by PRV infection in non-natural hosts has been frequently reported throughout the years. Currently, relatively few studies have focused on this particular aspect of PRV pathogenesis. This paucity of information is mainly because PRV remained a major viral disease in swine, causing substantial economic losses to pig producers. Therefore, the efforts of the research community were primarily focused on developing effective vaccines aimed at eradication of the virus. In this review, we highlight the more fundamental studies that focus on differences in the pathogenesis of PRV between pigs and non-natural hosts. These studies may explain the distinct clinical outcomes. Recently, researchers dissected the molecular and cellular mechanisms of PRV-induced neuropathic itch using several mouse models and emphasized the innate immune response as a central player. Good control of the inflammatory response during PRV infection of swine likely prevents the neuropathic pruritus experienced by infected non-natural hosts. Most importantly, PRV infection of mice has proven to be a suitable animal model to study PRV-induced neuropathic itch. This animal model may also provide useful insights into the pathogenesis of other herpesvirus infections, such as those following VZV infection. The model may lead to the development of innovative therapeutic strategies. Finally, this model may guide research on peripheral neuropathies such as multiple sclerosis and associated viral-induced damage to the PNS as well as other neurodegenerative processes.
